# Comparison of Pattern Electroretinography and Optical Coherence Tomography Parameters in Patients with Primary Open-Angle Glaucoma and Ocular Hypertension

**DOI:** 10.4274/tjo.39260

**Published:** 2015-12-05

**Authors:** Semra Tiryaki Demir, Mehmet Ersin Oba, Ezgi Tuna Erdoğan, Mahmut Odabaşı, Ayşe Burcu Dirim, Mehmet Demir, Efe Can, Orhan Kara, Selam Yekta Şendül

**Affiliations:** 1 Şişli Etfal Training and Research Hospital, Clinic of Ophthalmology, İstanbul, Turkey; 2 Kafkas University Faculty of Medicine, Department of Ophthalmology, Kars, Turkey; 3 İstanbul University İstanbul Faculty of Medicine, Department of Physiology, İstanbul, Turkey; 4 Medipol University Faculty of Medicine, Department of Ophthalmology, İstanbul, Turkey

**Keywords:** primary open-angle glaucoma, ocular hypertension, Retinal nerve fiber layer, ganglion cell complex, pattern electroretinogram

## Abstract

**Objectives:**

To investigate the correlation of visual field (VF), pattern electroretinography (PERG) and Fourier domain optical coherence tomography (FD-OCT) results in patients with ocular hypertension (OHT) and early primary open-angle glaucoma (POAG).

**Materials and Methods:**

The study included 72 eyes of 37 patients with early POAG, 76 eyes of 38 patients with OHT, and 60 eyes of 30 controls. All subjects underwent full ophthalmologic examination, VF assessment with 24-2 Humphrey standard automated perimetry (Swedish Interactive Thresholding Algorithm (SITA)-Standard), retinal nerve fiber layer (RNFL) and ganglion cell complex (GCC) thickness measurement with FD-OCT, and PERG P50 and N95 wave latency and amplitude measurements with electroretinography (Nihon Kohden).

**Results:**

With the exception of the nasal quadrant, all GCC parameters and RNFL results were significantly lower in the POAG group compared to the OHT and control groups. There was no statistically significant difference between the OHT and control group. PERG amplitudes were lower in the POAG and OHT groups than in the control group. Reduction in N95 amplitude was greater than that of P50 amplitude. No difference was detected in PERG latencies among groups. GCC was significantly correlated with VF and RNFL in the POAG group.

**Conclusion:**

Significant thinning of the GCC and RNFL occurs in addition to VF pathologies in patients with early POAG, and these examinations should be concomitantly evaluated. During diagnostic assessment of patients with early POAG, GCC and RNFL analysis by FD-OCT are highly effective. GCC is as reliable as RNLF in the early diagnosis of glaucoma and there is a highly significant correlation between them. Dysfunction of ganglion cells in patients with OHT may be detected earlier using PERG amplitude analysis.

## INTRODUCTION

Glaucoma is a progressive neuropathy and one of the leading causes of blindness worldwide. The nerve fiber damage that occurs in glaucoma is irreversible, making early diagnosis essential for its prevention. Ocular hypertension (OHT) is a clinical disorder in which intraocular pressure (IOP) is over 21 mmHg, yet no glaucomatous changes in the optic nerve head or visual field (VF) are found. Each year about 1% of patients diagnosed with OHT develop glaucomatous VF loss.^1^

Standard automated perimetry, accepted as the gold standard in the follow-up of glaucoma patients, is unable to detect defects if less than 40% of the ganglion cell axons are damaged.^2,3^ For this reason, investigators have sought new techniques for early diagnosis and follow-up. Optical coherence tomography (OCT) and pattern electroretinography (PERG) are currently used to supplement VF analysis in patients with primary open-angle glaucoma (POAG) and OHT.

OCT is a non-invasive method that provides high resolution cross-sectional tissue images. The retinal ganglion cells and nerve fiber layer are affected first in glaucoma. Recently, in addition to VF, Fourier domain OCT (FD-OCT) can measure retinal nerve fiber layer (RNFL) and ganglion cell complex (GCC) thickness, which are currently used in the diagnosis and treatment of glaucoma patients.^4,5^ PERG is used to objectively evaluate ganglion cell function in the macular region.^6,7,8^

There are few studies in the literature concerning the utility and correlation of VF, PERG, RNFL and GCC analyses in the diagnostic evaluation of POAG and OHT patients. Therefore, with this study we aimed to assess the utility of FD-OCT RNFL and GCC parameters and PERG latency and amplitude values in the diagnosis of early-stage POAG and OHT, and to assess how well these analyses correlate with VF and one another.

## MATERIALS AND METHODS

Patients diagnosed and followed in the Ophthalmology Clinic of Şişli Etfal Training and Research Hospital between January 2010 and August 2011 were enrolled prospectively for this controlled clinical study. Patients were separated into three groups comprised of 72 eyes of 37 early-stage POAG patients; 76 eyes of 38 OHT patients; and 60 eyes of 30 patients without glaucoma who visited the outpatient clinic for routine check-up as the control group. The study was approved by the local ethics committee. Patients were informed of the details of the study and all signed an informed consent form prior to their participation. The study was conducted in accordance with the principles of the Helsinki Declaration.

All patients underwent visual acuity assessment using the Snellen chart, biomicroscopic anterior and posterior segment examination, IOP measurement using Goldmann applanation tonometry, gonioscopic examination, and central corneal thickness measurement by ultrasonic pachymetry. Using Humphrey Field Analyzer II 750 (Zeiss Humphrey Systems) computerized automated perimetry, mean deviation (MD) and pattern standard deviation (PSD) values were evaluated by Swedish Interactive Thresholding Algorithm (SITA)-Standard 24-2 threshold test with false positive and false negative values under 30% and fixation loss under 20% considered reliable. Best corrected visual acuity was ≥0.7, spherical refraction was ±5 D, cylindrical refraction was ±2 D, and gonioscopy indicated open angle (grade III-IV by Shaffer classification) for all patients.

Patients who met the above criteria were placed in one of three groups. Group 1 comprised early-stage POAG patients (stage I: MD >6.00 dB, according to the Bascom Palmer [Hodapp-Anderson-Parrish] glaucoma staging system9) with untreated repeated IOP measurements over 21 mmHg and glaucoma-specific optic disc and VF damage. Group 2 comprised OHT patients with untreated repeated IOP measurements over 21 mmHg and normal optic disc, RNFL and VF. Group 3 was the control group and included cases with no ocular pathology, IOP under 21 mmHg, and normal optic disc, RNFL and VF.

For all patients, RNFL and GCC analyses were conducted using the RTVue-100 (Optovue, Inc., Fremont, CA, USA) device and PERG measurements were taken using a RM-6000 Nihon Kohden polygraph system. RNFL analysis included measurements of mean thickness, superior segment, inferior segment, and superior, inferior, temporal and nasal quadrant thickness values. GCC analysis included measurements of overall thickness, upper and lower quadrant thickness, as well as global loss volume (GLV) and focal loss volume (FLV). PERG measurements were conducted using silver skin electrodes. Recordings were done in accordance with the International Society for Clinical Electrophysiology of Vision (ISCEV) standards. For PERG recording, the active electrode was placed on the medial epicantus, while the reference electrode was placed in the temporal region of the same side, 2 cm from the eye. The ground electrode was placed on the opposite earlobe. Patients sat at a distance of 1 m from the screen; recordings were done as they looked at a fixation point on the screen with each eye while the other was closed. A checkerboard pattern of black and white contrasting squares was used for the PERG measurements. Stimulus frequency was 2 Hz and the analogue filter was between 0.03 and 100 Hz. Care was taken that the impedance remain under 5 kOhm. A total of 100 stimuli were used. Recordings were taken after the correction of any refractive errors. Analyses were done with the İstanbul Faculty of Medicine Electrophysiological Recording and Analysis System developed by Prof. Dr. Sacit Karamürsel. For PERG analysis, data obtained were filtered between 1 and 30 Hz. Artifacts in the data due to eye movements were eliminated individually. The amplitude and latency of PERG P50-N95 waves were evaluated. N95 wave amplitude was measured as the value from the P50 wave end to the N95 wave peak.

Patients with any of the following were excluded from the study: previous ocular surgery (other than uncomplicated cataract surgery), the appearance of narrow or closed angle in gonioscopic examination, fundus pathology (disc anomolies, macular pathology, retinal vascular diseases, etc.), pathologies leading to cloudy media (cataract, corneal pathology, etc.), secondary glaucoma (pseudoexfoliation, inflammation, trauma and lens-related conditions causing elevated IOP). Patients were also excluded if they had difficulty complying during the study or their test results did not indicate sufficient reliability.

SPSS version 13.0 (SPSS Inc., Chicago, USA) software package was used for statistical analyses. Intergroup parameter comparisons were made using one-way ANOVA, with p≤0.05 accepted as statistically significant. Tukey HSD test was used for pairwise comparisons and to determine the significance of intergroup differences. Pearson correlation analysis was used for the evaluation of correlations among parameters within groups.

## RESULTS

The study included 208 eyes of 105 patients. Comparisons of the study groups’ age, gender, VF, RNFL, GCC and PERG data are shown in Table 1. In the POAG group, average RNFL thickness values as well as values from the superior and inferior segments and the temporal, superior and inferior quadrants were significantly lower than in the OHT and control groups (p<0.05). Thickness values from the nasal quadrant showed no statistically significant difference between groups (p=0.096). The POAG group also exhibited significantly thinner GCC according to average, superior and inferior segment thickness values, while GLV and FLV were significantly greater (p<0.05). No significant differences emerged in GCC parameters between the OHT and control groups (p>0.05). PERG P50 and N95 wave amplitudes were significantly lower in the POAG and OHT groups compared to the control group (p<0.05). The decrease in N95 amplitude was greater than the decrease in P50 amplitude. There were no significant differences between groups in P50 and N95 wave latency values (p>0.05).

Correlation analyses of VF and RNFL, GCC and PERG values for the POAG and OHT groups are shown in Table 2. A significant correlation emerged between VF and GCC for the POAG group. A positive correlation was found between MD and inferior segment GCC thickness, and a negative correlation was found between MD and FLV (p<0.05).

Correlation analyses of RNFL and GCC parameters for the POAG and OHT groups are shown in Table 3. There was a significant correlation between RNFL and GCC parameters in the POAG group and in the OHT group, with the exception of the nasal quadrant (p<0.05). No correlation was found between PERG and RNFL or GCC in either group (p>0.05).

## DISCUSSION

The early diagnosis of glaucoma is critical for the prevention of irreversible nerve fiber damage. Retinal ganglion cell axon damage accounts for approximately 40% of early defects detected by standard automated perimetry, the gold standard for following glaucoma patients.^2,3^

Studies have shown that FD-OCT is reliable in the differentiation of glaucoma from normal eyes and in early diagnosis.^10^ It has also been shown that FD-OCT is consistent with VF analysis and useful as a complement to VF for identifying focal structural defects of the RNFL. It has been emphased that both RNFL and VF must be evaluated to detect early-stage glaucoma.^11,12,13^ Subbiah et al.^14^ found decreased RNFL thickness in glaucoma and OHT patients compared to normal cases. Studies have shown a correlation between VF and RNFL thickness in glaucoma patients, but not in OHT patients. In a study using Stratus OCT, Gyatsho et al.^15^ claimed that average and inferior quadrant RNFL thickness measurements are the best parameters for distinguishing glaucoma patients from the OHT and normal groups, whereas Taliantzis et al.^16^ demonstrated that average RNFL thickness measurement alone was inadequate for early-stage glaucoma and OHT patients. There are conflicting opinions among investigators regarding the utility of OCT in differentiating OHT patients from healthy cases. Garas et al.^5^ claims that OCT is effective and reliable for this purpose, while Schulze et al.^17^ deny its effectiveness. In Turkey, Sarıcaoğlu et al.^18^ found that the RNFL was markedly thinner in POAG patients compared to OHT and control groups, though there was no significant difference in thickness between the OHT and control groups.

Pierro et al.^19^ evaluated RNFL analyses by six different SD-OCT devices and observed differences in RNFL thicknesses between the devices. For this reason, they concluded that patients should be followed with the same device in order to obtain more accurate results.

In our study, patients in the POAG group exihibited significantly lower RNFL parameters compared to the OHT and control groups, with the exception of the nasal quadrant. Average thickness and superior and inferior quadrant thicknesses were especially low. According to these data, the nerve fibers in the superior and inferior quadrants are affected earliest in glaucoma, and these areas should be the focus of screening in patients suspected of developing glaucoma. In addition, as the temporal retina contains a higher density of retinal ganglion cells, we believe the fibers of the nasal quadrant are affected the latest. Therefore, we also believe that RNFL analysis by FD-OCT is reliable and effective in differentiating early-stage POAG and OHT from normal patients.

Histological studies have shown that ganglion cell density and standard perimetry findings are very closely associated.^20^ VF has been shown to be consistent with GCC.^21^ Recent studies have demonstrated that FD-OCT analysis of RNFL and GCC can be used in the diagnostic evaluation of glaucoma and OHT patients.^5^ Furthermore, marked thinning of the GCC has been reported in preperimetric glaucoma patients.^22^ Korkmaz et al.^23^ emphasized that GCC measurement is as important as RNFL measurement in the diagnosis and follow-up of POAG patients. Other investigators have stated that GCC is at least as reliable as RNFL in the early detection of glaucoma,^24^ and have found highly significant correlation between them.^25,26,27^ Akashi et al.^28^ used three different OCT instruments (Cirrus, RTVue and 3D-OCT) to compare early-stage glaucoma eyes with healthy eyes and demonstrated that all three can be used in the diagnostic evaluation of glaucoma.

The POAG patients in our study showed significantly lower values in GCC parameters compared with the OHT and control groups. For this reason, we also believe that FD-OCT analysis of GCC can be used to reliably and effectively distinguish early-stage POAG from OHT and normal patients. However, we did not detect significant differences between the OHT group and the control group in RNFL or GCC analysis, suggesting that FD-OCT is not sufficient or effective in the differentiation of OHT.

We found a significant correlation between VF and GCC in the POAG group. We believe VF and GCC should be evaluated together. In the OHT group, no correlation emerged between VF and GCC. However, in both the POAG and OHT groups, a significant correlation was found between RNFL and GCC, leading us to conclude that RNFL and GCC should be evaluated together.

Holder^6^ argued that ganglion cell damage could not be detected in OHT patients using available methods, and claimed that some of these patients are actually glaucoma patients who could not be diagnosed with glaucoma diagnostic methods. He also found that PERG could reveal signs of glaucoma progression in OHT patients. It has been shown that N95 amplitude is lower in OHT patients and that patients with high risk of developing glaucoma can benefit from PERG;^29^ glaucoma progression in OHT patients can be detected at least one year in advance using PERG.^30^ It has also been shown that glaucomatous damage can be detected earlier by PERG compared to standard automated perimetry.^31^ Falsini et al.^32^ found that decreased amplitude on PERG was consistent with RNFL thinning in early-stage glaucoma patients, but that RNFL thinning did not accompany the PERG amplitude decrease in OHT patients. Therefore, they concluded that PERG may be more beneficial than RNFL in the ganglion cell evaluation of OHT patients. PERG amplitudes correlate (though weakly) with VF, RNFL and GCC parameters,^33,34^ suggesting that PERG and OCT should be evaluated together.^35^

For the POAG and OHT patients in our study, evaluation of PERG amplitude was more valuable than latencies, and in particular the decrease in N95 amplitude was more pronounced. With PERG amplitude analysis, we believe that signs indicating the development of glaucoma in OHT patients can be detected at an earlier stage, before the appearance of VF and OCT findings. In contrast to the literature, in the current study we did not detect any correlations between PERG and VF, RNFL or GCC, which may be attributable to the wide range of normal values in PERG signal amplitudes.

In conclusion, POAG patients exhibit VF pathologies as well as marked RNFL and GCC thinning, and these evaluations should be performed concurrently. GCC and RNFL analysis by FD-OCT is effective for the diagnostic evaluation of early-stage POAG patients. GCC is at least as reliable as RNFL for the early diagnosis of glaucoma, and there is a strongly significant correlation between the two. In OHT patients, ganglion cell function loss can be detected in earlier stages using PERG amplitude analysis.

## Figures and Tables

**Table 1:**
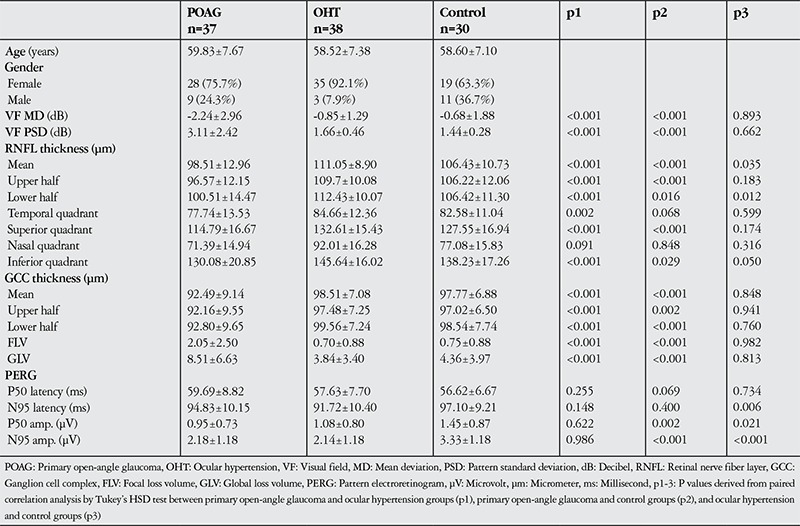
Comparison of the study groups’ age, gender, visual field, and retinal nerve fiber layer and ganglion cell complex thickness data acquired by pattern electroretinogram

**Table 2:**
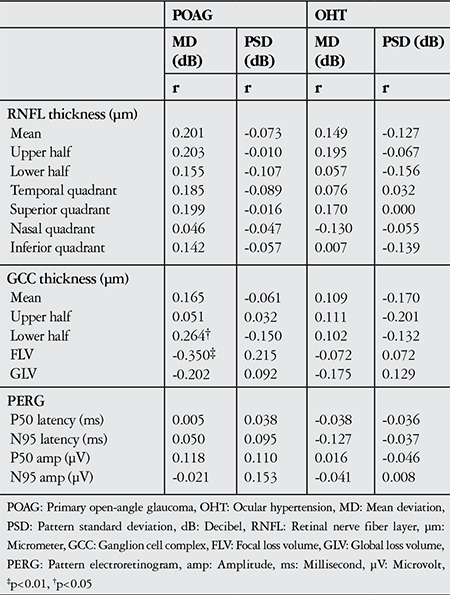
Correlation analysis of pattern electroretinogram visual field analysis and retinal nerve fiber layer and ganglion cell complex thickness parameters in primary open-angle glaucoma and ocular hypertension patients

**Table 3:**
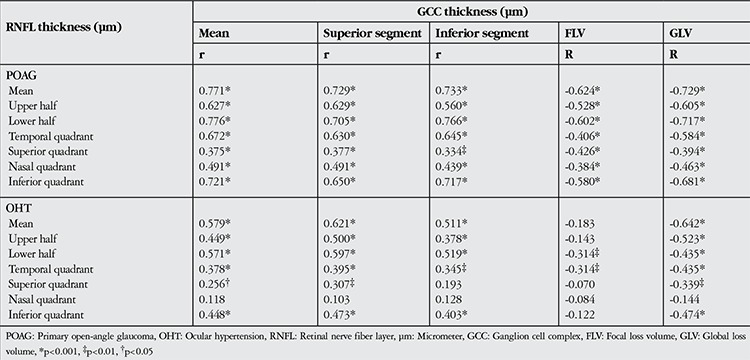
Correlation analysis of retinal nerve fiber layer and ganglion cell complex thickness values from the primary open-angle glaucoma and ocular hypertension groups
